# Efficacy of Electroacupuncture Combined with Auricular Point Pressing in Improving Mental Well-Being among Individuals with Heroin Use Disorder: A Randomized Controlled Crossover Trial and Pilot Study

**DOI:** 10.1155/2020/3748056

**Published:** 2020-09-25

**Authors:** Kai-Chiang Yu, Han-Ting Wei, Shang-Chih Chang, Chung-Hua Hsu

**Affiliations:** ^1^Department of Chinese Medicine, Taipei City Hospital, Linsen Chinese Medicine and Kunming Branch, Taipei, Taiwan; ^2^Institute of Traditional Medicine, National Yang-Ming University, Taipei, Taiwan; ^3^Department of Psychiatry, Taipei City Hospital, Linsen Chinese Medicine and Kunming Branch, Taipei, Taiwan

## Abstract

**Objective:**

To evaluate the clinical efficacy of combining electroacupuncture with auricular point pressing in improving quality of life of individuals with heroin use disorder undergoing methadone maintenance treatment.

**Design:**

A randomized controlled crossover trial.

**Subjects:**

50 participants were recruited from Taipei City Hospital, Linsen Chinese Medicine and Kunming branches, and randomly allocated to treatment groups.

**Method:**

The 36-Item Short Form Health Survey (SF-36) was used. Group A received electroacupuncture at the *Hegu* (LI4) and *Zusanli* (ST36) and auricular point pressing on Ear *Shenmen*, and Group B received only auricular point pressing on Ear *Shenmen* biweekly for 4 weeks. After a 1-week washout period, crossover of the groups was performed.

**Results:**

The SF-36 mental component scores of the combined treatment group improved relative to the single treatment group (11.09 vs. 10.33, *p*=0.023). Methadone dosage was reduced in both groups (combined therapy group: 8.58 ± 4.17/7.76 ± 4.11 (baseline/posttreatment) vs. single therapy group: 8.36 ± 4.20/8.30 ± .28, *p*=0.001).

**Conclusion:**

Combined therapy of high-frequency electroacupuncture with auricular point pressing had better efficacy in enhancing quality of life, especially for mental well-being, and in gradually reducing methadone dosage.

## 1. Introduction

Heroin use disorder is a prominent public health problem worldwide and a chronic medical illness. This disorder is associated with not only spreading of infectious diseases, such as HIV and hepatitis B and C, but also loss of productivity, disruption of personal relationships, crime, and violence [1]. However, opiate use disorders cause multiple types of damage to the normal functioning of individuals and thus require a multiaspect approach to the treatment process to improve their quality of life [2].

Treatment and prevention of heroin use disorder are a substantial public health concern [3]. Methadone and buprenorphine/naloxone are considered not only first-line treatments for heroin use disorder but also opioid substitution treatments. They can both alleviate opioid withdrawal symptoms and reduce opioid cravings, drug use, and mortality by reducing opioid use and improving quality of life [4, 5].

In 2006, an HIV/AIDS outbreak among people who use heroin intravenously in Taiwan had a considerable nationwide effect regarding heroin use and infectious disease control, and methadone maintenance treatment (MMT) was introduced [6, 7]. However, a multidisciplinary approach toward methadone must consider related clinical scenarios. Constipation, dizziness, drowsiness, headache, sweating, itchy skin, nausea, vomiting, and weakness are potential adverse effects of methadone [8].

Above all else, opiate use disorder is significantly associated with poor quality of life [2]. Individuals undergoing MMT may have poorer physical and psychological health than the general population, with a mental status similar to that of clinically depressed patients [9]. Approaches to improving the quality of life of individuals should include physiology and psychology [10, 11]. Low quality of life can easily lead to nonadherence to MMT [12].

The application of acupuncture has proven significant clinical effects on anxiety and depression, both physiologically and psychologically [13]. Patients undergoing MMT who received acupuncture treatment experienced significant improvement in quality of life, [14, 15] especially with treatment by biweekly application of electroacupuncture at *Hegu* on the hand, *Zusanli* on the leg, and *Shenmen* on the ear. With electroacupuncture as an adjunctive treatment for their withdrawal syndrome, these patients were able to reduce their methadone dosage [16, 17].

Electroacupuncture alleviates opioid withdrawal syndrome by releasing endogenous opioid neurotransmitters to relieve pain, along with exerting psychological effects after receptor binding [18, 19]. Electroacupuncture can stimulate neurotransmitters to bind with receptors, creating a cascade for a series of chemical reactions. However, low-frequency electroacupuncture (2 Hz) differs from high-frequency electroacupuncture (100 Hz) by stimulating the release of *β*-endorphin, endomorphins, and encephalins, which then interact with *µ*-opioid receptors and delta-opioid receptors. High-frequency electroacupuncture can accelerate the release of dynorphins, which bind to *κ*-opioid receptors [19–23].

Auricular point pressing treatment, an alternative to electroacupuncture, is an easily accessible adjunct therapeutic method for treating withdrawal symptoms during opiate detoxification. This treatment is beneficial in MMT for managing disorders related to drug use and follows theories and principles of Traditional Chinese Medicine (TCM) [24–27].

Reduction in quality of life related to poor physical and psychological health is closely associated with sleep quality in individuals who are dependent on heroin [28, 29].

In the treatment of individuals with opiate use disorders, improved quality of life is among the efficacies associated with both acupuncture and auricular point pressing. However, the efficacy of a combination treatment of electroacupuncture and auricular point pressing is unknown. Hence, the aim of this study was to evaluate the clinical efficacy of combined electroacupuncture and auricular point pressing in improving the quality of life of individuals with heroin use disorder undergoing MMT.

## 2. Materials and Methods

### 2.1. Study Participants

The study was conducted from June 23, 2017, to December 28, 2018. We invited 72 individuals in clinics of the Linsen Chinese Medicine and Kunming branches of Taipei City Hospital (Taipei, Taiwan), which offers standard methadone therapy, to participate.

Inclusion criteria were as follows: (1) the participant was between 20 and 65 years of age and (2) received a diagnosis of heroin disorder based on the Diagnostic and Statistical Manual of Mental Disorders, Fifth Edition. Exclusion criteria involved the following: (1) pregnancy, (2) refusal to receive electroacupuncture or auricular point pressing treatment, (3) appearance of major physical or mental disorders, and (4) apparently irregular or fluctuating dosage of methadone or apparent morphine withdrawal syndrome. During their first clinic visit, participants were interviewed by a research assistant and signed informed consent forms approved by the Research Ethics Committee of Taipei City Hospital (IRB number: TCHIRB-10601106). Baseline assessments were conducted in Taipei City Hospital, Linsen Chinese Medicine and Kunming branches, clinics. The trial was registered with ClinicalTrials.gov (Identifier: NCT03881618).

### 2.2. Randomization

After completion of the baseline assessment, all eligible participants received a sealed envelope containing a computer-generated random number collected by the author who did not have direct contact with participants. Allocation was concealed until completion of all baseline assessments. Participants were randomly allocated to one of the two groups in a 1 : 1 ratio (Group A, *n* = 25 and Group B, *n* = 25) and started receiving treatment according to their assignment.

### 2.3. Interventions

Acupuncture points for the electroacupuncture intervention were selected on the basis of studies examining the efficacy of acupuncture for the treatment of opioid addiction [13, 16, 17, 30–33]. *Hegu* and *Zusanli* were chosen as two acupoints. They consisted of four bilateral acupuncture points on the hands and legs, and electroacupuncture was administered using an electroacupuncture machine (Model D0310K, Ching Ming Medical Device Co. Ltd., Taiwan) delivering a frequency of 100 Hz (dense and disperse, DD). Electroacupuncture was dispersed at automatic 2-second intervals for 20 minutes to lead to “DeQi” (revealed as numbness, soreness, and heaviness), with increasing 1 mA increments of intensity to the maximum tolerable intensity as reported by the participant. In addition, bilateral Shenmen auricular acupoints were fixed with cowherb seed by a breathable tape and stimulated by manual pressing every five minutes for a total of 20 minutes, in accordance with relevant research [24–26]. All TCM interventions and care were provided in quiet, private clinics of the Linsen Chinese Medicine and Kunming branches of Taipei City Hospital, Taipei, Taiwan, by the same attending clinician with 10 years of acupuncture experience, and all interventions were performed in the evening (between 5:00 and 8:00 pm). The needles and cowherb seeds were removed immediately afterward by the same clinician after intervention.

### 2.4. Study Process

After the exclusion of ineligible individuals, 50 (69%) patients aged between 20 and 65 years were included in the parallel intervention. Group A received combined therapy (electroacupuncture at the *Hegu* (LI4) and *Zusanli* (ST36) and auricular point press on Ear *Shenmen*), whereas Group B received a single therapy (auricular point press on Ear *Shenmen*). The intervention was performed biweekly for 4 weeks (Stage I treatment, days 1–28). After a 1-week washout period (days 29–35), a crossover of the groups was performed (Stage II treatment, days 36–64). During both treatments, all participants received treatment biweekly for 4 weeks continually and without interruption.

### 2.5. Outcome Measurement

Outcome measurements of the treatments on days 1, 28, 36, and 64 were made using the Taiwanese version of the 36-Item Short Form Health Survey (SF-36) to evaluate quality of life at the completion of treatment courses. The Taiwanese version of the instrument demonstrated validity similar to that of other language versions [34]. To evaluate quality of life, eight domains are assessed: physical functioning (PF), role limitations due to physical problems (RP), bodily pain (BP), general health (GH), vitality (VT), social functioning (SF), role limitations due to emotional problems (RE), and general mental health (MH) [35]. Five of the scales (PF, RP, BP, SF, and RE) describe limitations or disability, with the remaining three (GH, VT, and MH) denoting a state of welfare where midrange scores indicate no limitations or disabilities. For this study, items in each domain were aggregated and transformed into a scale from 0 to 100, with higher scores indicating better health status [34–36]. Moreover, two distinct concepts are measured by the SF-36: a physical dimension, represented by a physical component summary (PCS) containing PF, RP, BP, and GH scores, and a mental dimension, represented by a mental component summary (MCS) [37] containing VT, SF, RE, and MH scores.

In methadone maintenance treatment, opioid withdrawal syndrome may be observed among patients with irregular adherence, causing poor quality of life. Therefore, a gradual and smooth methadone dosage tapering without heroin relapse is inevitably a vivid goal for individuals with heroin use disorder.

### 2.6. Sample Size

Sample size was calculated using the following input parameters: two tails, an error probability = 0.05, and power (1 − *b* error probability) = 0.8. The required size was calculated to be 35. With a 20% dropout rate, approximately 44 participants were required.

### 2.7. Statistical Analysis

Statistical analysis was performed by another researcher who was unaware of treatment allocation. Demographic and other data for age, sex, occupation, marital status, and history of methadone use, hepatitis B, hepatitis C, HIV, and syphilis were collected and analyzed using SPSS (version 22.0). The demographic characteristics were first analyzed using descriptive statistics, including the mean, standard deviation, and percentage. Inferential statistics, including the independent *t*-test, were used to verify homogeneity and the effect of electroacupuncture among groups. The paired *t*-test was used to evaluate within-group differences. Statistical significance was set at a *p* value of 0.05, two-tailed.

## 3. Results

**Figure 1:**
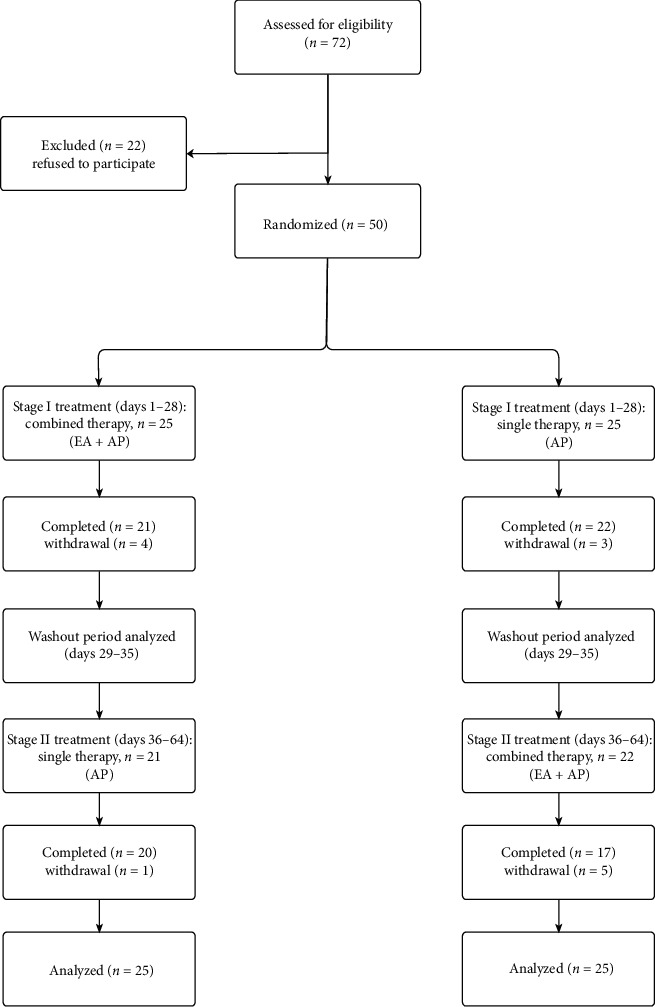
Participant flowchart.

Figure 1 presents the study flowchart. Among 72 eligible participants, 22 were excluded as they did not wish to try acupuncture. In total, 50 participants were enrolled, of whom 37 completed both stages of the study. During the study period, five participants (20%) in Group A dropped out. Four were afraid of needle treatment in Stage I and one participant was unwilling to accept Stage II treatment. Eight participants in Group B (32%) dropped out (three because they were unwilling to complete the questionnaire at Stage I and five because they were afraid of needle treatment at Stage II). Demographic data revealed no significant differences between these groups (Table 1).

### 3.1. Treatment Outcome on Quality of Life

Sizeable treatment outcomes were noted in Group A (*n* = 25)/Stage I and Group B (*n* = 25)/Stage II. A significant improvement through combined therapy was observed in the RP (Group B/Stage I and Group B/Stage II), BP (Group A/Stage I and Group B/Stage I), VT (Group A/Stage I and Group B/Stage I), RE (Group B/Stage II), and MH (Group B/Stage II) subscales (Table 2).

We divided data for treatment groups for comparison (Table 3). Significant differences in vitality (VT: *p* *=* 0.012) and mental health (MH: *p* < 0.001) subscales were observed.

**Figure 2:**
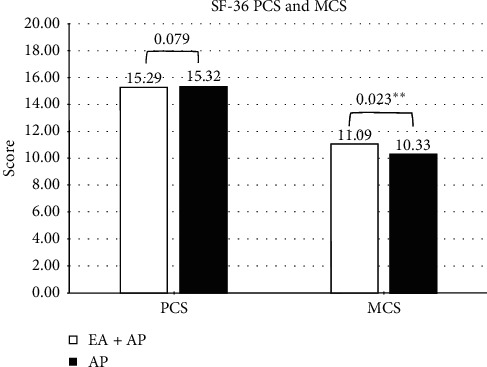
Physicalcomponentscore (PCS) and mental component score (MCS) of combined therapy group and single therapy group. Note: ^*∗*^*p* < 0.05; ^*∗∗*^*p* < 0.01; ^*∗∗∗*^*p* < 0.001.

Data collected using the SF-36 were also analyzed by the physical component score (PCS) and mental component score (MCS) for evaluation. We uncovered a significantly higher percentage of improvement in the MCS score of the combined treatment group vs. the single treatment group (11.09 vs. 10.33, *p* *=* .023; Figure 2).

### 3.2. Methadone Dosage of Participants

We closely monitored methadone dosage among participants (Table 4). Dosage significantly decreased in both the combined (baseline vs. posttreatment: 8.32 ± 4.11 vs. 7.56 ± 3.91, *p* = 0.002) and single (8.27 ± 3.97 vs. 7.97 ± 4.15, *p* = .027) treatment groups.

### 3.3. Adverse Reactions

One participant presented with mild bleeding at the acupuncture point after Group B Stage II treatments. Another participant reported a feeling of dizziness after Group A Stage I treatments. Immediate supportive medical treatments were provided and followed to conclusion.

## 4. Discussion

Individuals with heroin use disorder may experience heroin relapse. Therefore, relapse prevention approaches that improve the physical and mental health and well-being of individuals with heroin use disorder are vital during recovery. A low quality of life during MMT may be a latent risk factor for relapse [3]. Furthermore, the larger the amount of opiate used during MMT, the more severe the withdrawal syndrome symptoms are—especially its psychological symptoms [9]. A goal of improvement in client quality of life is decreased methadone dosage. When dosage is reduced gradually, withdrawal is less severe, creating a virtuous cycle with improved quality of life [3].

Our results indicate that combined therapy resulted in a larger decline in dosages (Table 4), which would also indirectly reduce the incidence of withdrawal syndrome through improving quality of life [38]. This improvement was more evident in MCS scores (combined therapy: 11.09 vs. single therapy: 10.33, *p* *=* 0.023) than in PCS scores (combined therapy: 15.29 vs. single therapy: 15.32, *p* *=* 0.079; Figure 2). The largest effects were observed in VT (combined therapy baseline : posttreatment (12.00 ± 1.90 : 13.29 ± 2.31) vs. single therapy baseline : posttreatment (11.78 ± 2.29 : 12.54 ± 2.04), *p* *=* .012) and RE (combined therapy: 4.45 ± 0.90 vs. single therapy: 4.97 ± 0.64, *p* *=* 0.007) subscales.

The mechanism of electroacupuncture has been revealed to be associated with opioid receptors (kappa, delta, and mu) in brain regions known to interact with endogenous opioids and that are believed to play an essential role in life quality [39, 40]. Exogenous opioids, such as morphine, bind to the same sites as endogenous opioids, suppressing the production of endogenous opioids. Withdrawal syndrome may appear when morphine exposure is suddenly stopped [41, 42]. Acupuncture can compensate for the lack of a chemical cascade from neurotransmitters.

Auricular point pressing and electroacupuncture treatment follow theories and principles of TCM to improve Chi circulation [43]. Auricular point pressing was included in the design of both interventions because it is convenient and accessible at home by clients themselves, whereas invasive treatment must be executed at a clinic.

A partially single adjunct therapeutic method to treat withdrawal symptoms during opiate detoxification has been beneficial in MMT in managing related disorders [23–26]. A daily practice of appropriate auricular point pressing may also strengthen efficacy for improving RP (single therapy, baseline: 7.27 ± 1.40 vs. posttreatment: 7.70 ± 0.93, *p* *=* 0.021), GH (single therapy, baseline: 15.37 ± 1.08 vs. posttreatment: 15.91 ± 1.11, *p* *=* 0.044), and VT (single therapy, baseline: 11.78 ± 2.29 vs. posttreatment: 12.54 ± 2.04, *p* *=* 0.011; Table 3) subscales. Adding electroacupuncture also added efficacy according to MCS results (combined therapy: 11.09 vs. single therapy: 10.33, *p* *=* 0.023). Maintaining mental health is the most difficult challenging aspect of MMT for individuals with heroin disorder [9].

Little research has been conducted to study the clinical effect of high-frequency electroacupuncture. All that is known is that 100 Hz was more effective than 2 Hz in an animal study, owing to the promotion of endogenous opioids, such as dynorphin, which binds to kappa opioid receptors to ameliorate withdrawal syndrome. We demonstrated that high-frequency electroacupuncture reduced withdrawal syndrome among clients undergoing MMT as measured by SF-36, especially its mental domains. Furthermore, it may result in declining methadone dosage (Table 4).

Because auricular point pressing is a noninvasive, evidence-based, access-friendly TCM treatment, the related treatment program should be addressed by addiction specialists. TCM treatments are widely accepted in Taiwanese and Chinese culture, whether electroacupuncture or auricular point pressing. Auricular point pressing in a TCM clinic with adequate supervision by trained TCM clinicians improves quality of life, and combining it with high-frequency electroacupuncture further improves outcomes. Finally, for ethical reasons, we did not include a sham treatment [24–26] for participants.

The clinical impact of the study is in its demonstration that TCM treatments, especially electroacupuncture with auricular point pressing, can be introduced as a routine facilitating treatment for patients undergoing MMT.

### 4.1. Limitations

This study has several limitations. First, it included a small number of participants. However, through a randomized crossover controlled design, we could gather data with sufficient statistical power. Second, this was an open-label study and thus lacked blinding. Third, the washout period was short, largely because participants undergoing MMT belonged to a particular ethnic group, making tracing and evaluation with a longer washout period too risky.

## 5. Conclusion

A combined therapy of electroacupuncture and auricular point pressing is more effective than only auricular point pressing in improving the quality of life of individuals with heroin use disorder undergoing MMT. TCM treatments, particularly electroacupuncture with auricular point pressing, can be introduced as a routine adjunctive treatment.

## Figures and Tables

**Table 1:** Demographic and clinical characteristics of participants.

Variable definition	Group A (*n* = 25)	Group B (*n* = 25)	Total (*n* = 50)	*p* value
Age (years)	46.20 ± 8.09	45.47 ± 7.85	45.99 ± 7.83	0.783
Sex				0.508
Male	18 (72.0)	16 (64.0) 9 (36.0)	34 (68.0)	
Female	7 (28.0)		16 (32.0)	
Occupation				0.970
Employed	18 (72.0)	17 (68.0)	35 (70.0)	
Unemployed	7 (28.0)	8 (32.0)	15 (30.0)	
History of methadone use (years)	6.70 ± 3.21	7.18 ± 3.09	6.94 ± 3.12	0.650
Hepatitis B				0.860
Positive	5 (20.0)	4 (16.0)	9 (18.0)	
Negative	20 (80.0)	21 (84.0)	41 (82.0)	
Hepatitis C				0.868
Positive	23 (92.0)	22 (88.0)	45 (90.0)	
Negative	2 (8.0)	3 (12.0)	5 (10.0)	
HIV				0.228
Positive	2 (8.0)	5 (20.0)	7 (14.0)	
Negative	23 (92.0)	20 (80.0)	43 (86.0)	
Syphilis				0.284
Positive	0 (0.0)	2 (8.0)	2 (4.0)	
Negative	25 (100.0)	23 (92.0)	48 (96.0)	
Marital status				0.463
Never married	9 (36.0)	9 (36.0)	18 (36.0)	
Married/cohabiting	9 (36.0)	15 (60.0)	24 (48.0)	
Divorced/widowed	7 (28.0)	1 (4.0)	8 (16.0)	

**Table 2:** SF-36 results at Stage I and Stage II.

Measurements	Stage	Days	Group A mean (SD)	Group B mean (SD)	*p* value
SF-36					
*Physical functioning (PF)*	I	0	29.15 (2.36)	28.29 (2.97)	
	I	28	29.25 (2.35)	28.58 (2.95)	0.610
*p* value for paired *t*-test			0.163	0.236	
	II	36	29.15 (2.34)	28.52 (2.60)	
	II	64	29.20 (2.30)	28.35 (2.59)	0.433
*p* value for paired *t*-test			0.330	0.332	
*Role limitation due to physical problems (RP)*	I	0	7.30 (1.34)	7.00 (1.65)	
	I	28	7.75 (0.71)	7.76 (0.97)	0.854
*p* value for paired *t*-test			0.095	0.049^*∗*^	
	II	36	7.50 (1.14)	6.58 (1.58)	
	II	64	7.65 (0.93)	7.52 (0.94)	0.826
*p* value for paired *t*-test			0.186	0.013^*∗*^	
*Bodily pain (BP)*	I	0	9.45 (1.98)	8.58 (2.29)	
	I	28	10.10 (1.29)	9.58 (1.46)	0.496
*p* value for paired *t*-test			0.015^*∗*^	0.036^*∗*^	
	II	36	9.40 (1.69)	8.52 (1.66)	
	II	64	9.35 (1.66)	8.88 (1.69)	0.574
*p* value for paired *t*-test			0.863	0.138	
*General health (GH)*	I	0	15.70 (0.97)	15.05 (0.82)	
	I	28	16.10 (0.71)	15.55 (0.62)	0.511
*p* value for paired *t*-test			0.189	0.120	
	II	36	15.75 (0.71)	15.52 (1.00)	
	II	64	15.55 (0.94)	15.52 (0.71)	1.000
*p* value for paired *t*-test			0.479	1.000	
*Vitality (VT)*	I	0	12.60 (1.95)	11.52 (2.57)	
	I	28	14.25 (1.86)	12.88 (2.11)	0.018^*∗*^
*p* value for paired *t*-test			0.002^*∗*^	0.030^*∗*^	
	II	36	12.00 (2.07)	11.29 (1.61)	
	II	64	12.25 (1.99)	12.17 (2.35)	0.586
*p* value for paired *t*-test			0.096	0.060	
*Social function (SF)*	I	0	6.95 (1.39)	6.64 (1.45)	
	I	28	7.25 (1.61)	6.88 (1.49)	0.459
*p* value for paired *t*-test			0.527	0.637	
	II	36	7.00 (1.25)	7.00 (1.45)	
	II	64	7.20 (1.39)	7.05 (1.43)	0.569
*p* value for paired *t*-test			0.494	0.896	
*Role limitation due to emotional problems (RE)*	I	0	4.60 (1.09)	4.41 (1.00)	
	I	28	5.00 (0.72)	4.64 (0.93)	0.231
*p* value for paired *t*-test			0.202	0.482	
	II	36	4.65 (0.48)	4.29 (0.58)	
	II	64	4.70 (0.65)	4.94 (0.55)	0.455
*p* value for paired *t*-test			0.772	0.002^*∗∗*^	
*Mental health (MH)*	I	0	20.50 (1.82)	17.23 (1.56)	
	I	28	19.95 (1.27)	17.58 (1.46)	<0.001^*∗∗∗*^
*p* value for paired *t*-test			0.290	0.269	
	II	36	17.70 (2.07)	20.17 (1.70)	
	II	64	17.85 (2.10)	18.94 (1.29)	0.256
*p* value for paired *t*-test			0.697	0.012^*∗*^	

*Note*. SF-36 = 36-Item Short Form Health Survey; ^*∗*^*p* < 0.05; ^*∗∗*^*p* < 0.01; ^*∗∗∗*^*p* < 0.001.

**Table 3:** SF-36 results at baseline and posttreatment (electroacupuncture plus auricular point pressing vs. auricular point pressing alone).

	Electroacupuncture ＋ auricular point pressing		Auricular point pressing		*p* value
	Mean (SD)	Paired *t*-test *p* value	Mean (SD)	Paired *t*-test *p* value	
*Physical functioning (PF)*					
Baseline	28.86 (2.46)	0.768	28.75 (2.65)	0.160	0.975
Posttreatment	28.83 (2.47)		28.91 (2.60)		
*Role limitations of physical problems (RP)*					
Baseline	6.97 (1.48)	0.003^*∗∗*^	7.27 (1.40)	0.021^*∗*^	0.358
Posttreatment	7.64 (0.82)		7.70 (0.93)		
*Bodily pain (BP)*					
Baseline	9.02 (1.87)	0.004^*∗∗*^	9.02 (2.00)	0.111	0.893
Posttreatment	9.54 (1.59)		9.45 (1.55)		
*General health (GH)*					
Baseline	15.48 (1.04)	0.026^*∗∗*^	15.37 (1.08)	0.044^*∗*^	0.846
Posttreatment	16.08 (1.21)		15.91 (1.11)		
*Vitality (VT)*					
Baseline	12.00 (1.90)	<0.001^*∗∗∗*^	11.78 (2.29)	0.011^*∗∗*^	0.012^*∗∗*^
Posttreatment	13.29 (2.31)		12.54 (2.04)		
*Social function (SF)*					
Baseline	6.97 (1.40)	0.557	6.83 (1.34)	0.427	0.611
Posttreatment	7.16 (1.51)		7.05 (1.43)		
*Role limitations due to emotional problems (RE)*					
Baseline	4.45 (0.90)	0.007^*∗∗*^	4.54 (0.76)	0.442	0.437
Posttreatment	4.97 (0.64)		4.67 (0.78)		
*Mental health (MH)*					
Baseline	20.35 (1.75)	0.015^*∗*^	17.48 (1.85)	0.330	<0.001^*∗∗∗*^
Posttreatment	19.48 (1.36)		17.72 (1.82)		

*Note*. ^*∗*^*p* < 0.05; ^*∗∗*^*p* < 0.01; ^*∗∗∗*^*p* < 0.001.

**Table 4:** Methadone dosage at baseline and posttreatment (electroacupuncture plus auricular point pressing vs. acupressure point pressing alone).

	Electroacupuncture ＋ auricular point pressing		Auricular point pressing			
	Mean (SD)	Paired *t*-test *p* value	Mean (SD)	Paired *t*-test *p* value	*p* value	
Dosage						
Baseline (mg/day)	8.32 (4.11)	0.002^*∗∗*^	8.27 (3.97)	0.027^*∗*^	0.001^*∗∗∗*^	
Posttreatment (mg/day)	7.56 (3.91)		7.97 (4.15)			

	Mean (SD)		Mean (SD)		*p* value	Mean difference (95% CI)

Posttreatment-baseline (mg/day)	−0.75 (4.01)		−0.30 (4.06)		0.043^*∗∗*^	−0.83, −0.27

*Note*. ^*∗*^*p* < 0.05; ^*∗∗*^*p* < 0.01; ^*∗∗∗*^*p* < 0.001.

## Data Availability

The data used to support the findings of this study are available from the corresponding author upon request.

## Abbreviations

MMT: Methadone maintenance treatment
TCM: Traditional Chinese Medicine
MCS: Mental component summary of the SF-36
PCS: Physical component summary of the SF-36
PF: Physical functioning
RP: Role limitation due to physical problems
BP: Bodily pain
GH: General health
VT: Vitality
SF: Social function
RE: Role limitation due to emotional problems
MH: Mental health.
